# Effectiveness of Multiple Mini Interviews in medical school admissions: assessment using The Big Five Personality framework

**DOI:** 10.31744/einstein_journal/2025AO1352

**Published:** 2025-04-25

**Authors:** João Sampaio Góes, Stefano Ivani de Paula, Durval Aníbal Daniel-Filho, Elda Maria Stafuzza Gonçalves Pires, Ângela Tavares Paes, Eduardo Juan Troster

**Affiliations:** 1 Faculdade Israelita de Ciências da Saúde Albert Einstein Hospital Israelita Albert Einstein São Paulo SP Brazil Faculdade Israelita de Ciências da Saúde Albert Einstein, Hospital Israelita Albert Einstein, São Paulo, SP, Brazil.; 2 Hospital Israelita Albert Einstein São Paulo SP Brazil Hospital Israelita Albert Einstein, São Paulo, SP, Brazil.

**Keywords:** Medical education, Personality tests, School admission criteria, Medical schools, Interview

## Abstract

**Objective:**

To evaluate the effectiveness of Multiple Mini Interviews as a selection method for medical school admissions at the Faculdade Israelita de Ciências da Saúde Albert Einstein by evaluating the relationship between the Big Five personality traits and the performance of candidates in the Multiple Mini Interviews.

**Methods:**

All candidates who had completed the NEO PI-R personality test and the socio-demographic questionnaire were the participants of the study. The personality profiles of candidates who participated in the Multiple Mini Interviews process of this cross-sectional descriptive study were analyzed by crossing data to identify significant correlations.

**Results:**

Of the 225 candidates, 63% were female and the mean age of the sample was 21 years. Only the domain of Conscientiousness showed significant correlations with higher Multiple Mini Interviews scores (p=0.004). Specifically, traits of Conscientiousness such as Competence (p=0.046), Dutifulness (p=0.043), Achievement-Oriented (p=0.050) and Self-Discipline (p=0.028) were associated with better Multiple Mini Interviews performance. Other domains, like Neuroticism, Extraversion, Openness, and Agreeableness, showed no significant correlation. Additionally, socio-demographic factors had no significant impact on performance.

**Conclusion:**

The findings validate the Multiple Mini Interviews as an effective method for medical school selection, as they correlate with high-value personality traits such as Conscientiousness, while not favoring irrelevant individualities. These results orient the enhancement of selection methods, ensuring a diverse and competent student body and, overall, improving medical education. The findings thus support the use of structured interviews in admission processes, highlighting their role in identifying candidates with essential qualities for success in medical education and practice, and offer valuable insights for similar educational institutions aiming to refine their selection methods.

## In Brief

This study highlights how the Multiple Mini Interviews can be an effective format to evaluate the socio-emotional competencies of candidates seeking admission to medical school. Candidates with higher levels of conscientiousness, revealing traits of competence and self-discipline, achieved superior performance. These traits are linked to future professional success.

## Highlights

■ Multiple Mini Interviews assess medical school candidates’ non-cognitive skills effectively.

■ Conscientiousness is found to strongly correlate with higher Multiple Mini Interviews performance.

■ Multiple Mini Interviews ensure diversity without bias toward irrelevant personality traits. ■ Structured interview formats predict academic success and professional readiness.

## Graphical abstract

**Figure:**
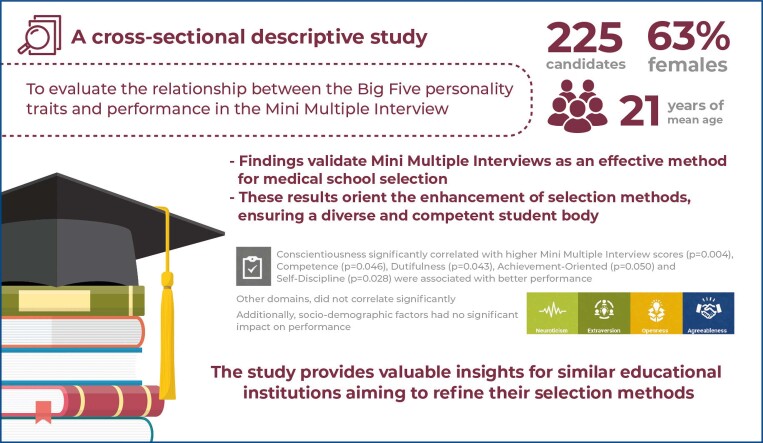

## INTRODUCTION

Healthcare professionals need to possess a combination of scientific knowledge, an ethical stance, and technical and behavioral skills in their daily practice. Achieving professional competence in the medical field necessitates not only cognitive abilities but also a robust socio-emotional skillset.^1^

For effective practice in the healthcare profession, decision-making, leadership, teamwork, empathy, professionalism, and respect for diversity are critical.^1^ Given that these non-cognitive skills are gaining increasing recognition, many medical schools worldwide have incorporated evaluation of socio-emotional skills into their selection processes to ensure that future medical practitioners possess the necessary attributes for high-quality practice.^2 , 3^

Consequently, a growing interest has been noticed in methods to assess these attributes in the medical school admissions process. Traditionally, individual and standardized interviews were used for evaluation, but this process often lacked validity, reliability, and reproducibility.^4 , 5^

Structured interviews, such as Multiple Mini Interviews (MMIs), have emerged as an effective instrument for assessing socio-emotional skills.^6 , 7^ In MMIs, candidates are evaluated across a series of stations designed to assess their ability to think logically and ethically and communicate effectively.^7 , 8^ Systematic reviews have shown MMIs to be cost-effective, efficient, and fair, without demonstrating socioeconomic, cultural, or gender biases. Furthermore, scenario-based stations reduce the likelihood of candidates preparing standardized responses, thus leading to a more genuine assessment of their abilities.^7^

More importantly, MMI performance has been shown to have no correlation with prior academic performance, suggesting that the MMI assesses different competencies. Some studies even indicate that MMI scores may predict future academic success in medical school.^7 , 9 , 10^

Recent research has explored the relationship between work performance and personality traits,^11 - 13^ with many using the Big Five model as a framework for evaluating personality profiles.^14^ The Big Five model encompasses five major domains: Neuroticism, Extraversion, Openness, Agreeableness, and Conscientiousness. Each domain comprises several facets that provide a comprehensive view of an individual’s personality.

Neuroticism: Anxiety, Anger, Depression, Embarrassment, Impulsiveness, Vulnerability; Extraversion: Warmth, Gregariousness, Assertiveness, Activity, Excitement-Seeking, Positive Emotions; Openness: Fantasy, Aesthetics, Feelings, Actions, Ideas, Values; Agreeableness: Trust, Straightforwardness, Altruism, Compliance, Modesty, Compassion. Conscientiousness: Competence, Orderliness, Dutifulness, Achievement-Oriented, Self-Discipline, Deliberation.^14^

According to McCrae,^15^ personality traits are global and abstract dispositions that summarize the tendencies, styles, and preferences of individuals rather than their concrete behaviors.^15 , 16^ Some authors argue that contextual factors rather than stable personality traits influence socio-emotional reactivity.^17^

Diversity in the academic environment enhances learning, fosters scientific creativity, and facilitates personal and professional growth.^18 , 19^ Therefore, MMIs are meant to evaluate socio-emotional skills without favoring irrelevant personality profiles. This study clarifies whether, in the Brazilian cultural context, MMI are equanimous in discriminating while selecting desirable personality attributes.^20^Despite the controversy in the literature about the correlation between the Big Five and MMI, no reports of analyses regarding the personality profiles of students subjected to this method have been found from Brazil.^21 - 23^

## OBJECTIVE

Using the Big Five personality traits as a conceptual framework, this study aims to evaluate how the MMI functions as an effective selection tool for medical school admissions. Specifically, it examines whether the MMI selects students with diverse personality profiles or tends to prioritize certain traits. In doing so, this research provides a deeper understanding of the selection process and offers valuable insights for future studies on medical school admissions.

## METHODS

### Study design

This cross-sectional study is designed to explore the relationship between the Big Five personality traits and performance in the MMI used in the medical school selection process at the *Faculdade Israelita de Ciências da Saúde Albert Einstein* in 2021. The sample included all candidates who applied to the medical program and passed the initial phase of the entrance examination.

The selection process was structured into two distinct phases. The first phase is a theoretical knowledge test comprising 50 multiple-choice questions, each with four alternatives, covering all basic school disciplines; and five dissertation questions and one critical thinking essay. Among the candidates who wrote the test, only the top 256 candidates in the first phase ranking proceeded to the second phase. The second phase involves the MMIs, conducted across eight stations. At each station, candidates are given 2 minutes to read a scenario and 6 minutes to respond and interact. During these interactions, a diverse set of situations are presented to them, and they are evaluated on a scale of 1 to 7, on themes such as Compassion, Empathy, Effective Communication, Ethics, Leadership, Critical Thinking, and Teamwork.

In both phases, raw scores were standardized using the Item Response Theory. Two rankings were created based on the scores: MMI Scores Only and Combined data. The first indicated ranking based solely on candidates’ MMI scores. The second indicated ranking based on the combined scores of the first and second phases, weighted 75% and 25%, respectively. The top 63 candidates in the combined scores ranking were admitted in the first call.

### Data collection

Two questionnaires were administered to 225 subjects: Socio-Demographic Questionnaire: Candidates had to answer this during registration and provide information regarding their age, sex, marital status, family residence location, academic background, parental education levels, and family income ( Appendix 1 ). NEO PI-R Questionnaire: The NEO PI-R, exclusively used by psychologists, consists of 240 items, evaluates six facets within each of the five Big Five domains: Neuroticism, Extraversion, Openness, Agreeableness, and Conscientiousness ( Appendix 2 ).

To avoid measurement bias, the questionnaires were emailed to the candidates a week after they completed the MMI and before the official results were announced. This was done to ensure that the admissions process was not influenced by the scores.

After the candidates completed the online questionnaires, Vetor Editora, which conducts the test in Brazil, received all responses and provided individual reports analyzing the five domains and thirty facets. Only candidates who completed both the NEO PI-R and socio-demographic questionnaires were included in the study. Those who did not sign the informed consent form or provided incomplete questionnaire responses were excluded.

### Statistical analysis

In the descriptive analysis, quantitative variables were summarized using medians, standard deviations, and minimum and maximum values. Qualitative variables were summarized using relative and absolute frequencies.^24^Data normality was assessed using descriptive statistics, histograms, box plots, and normal probability plots. Homoscedasticity was verified using Levene’s test, and when this assumption was violated, the Brown-Forsythe correction was applied.

Candidates were categorized into those “Admitted in the 1st call” and those “Not admitted in the 1st call.” Comparative analyses of personality profiles between candidates with higher and lower scores were performed using ANOVA for normally distributed data. The personality profile of the participants was characterized by describing the scores of the facets and domains of the NEO PI-R instrument.

Additionally, the direct relationship between MMI scores and the NEO PI-R scores was analyzed using Pearson’s linear correlation coefficients. All analyses were conducted using Jamovi version 2.3.21, R version 4.3.0, and SPSS version 19.0 software. The significance level was set at 5%.

### Ethical considerations

This study was submitted to the Research Ethics Committee of the *Hospital Israelita Albert Einstein* (CAAE: 37755620.5.0000.0071; 4.320.241). To avoid conflicts of interest, none of the researchers was involved in the admission process. Students invited to participate in the research were informed about its objectives and importance. Those willing to participate received and signed the Informed Consent Form before completing the questionnaires.

### Risks and benefits

Potential risks included breach of confidentiality. To mitigate this, all research instruments were identified only by an alphanumeric code, avoiding personal identification. These measures were aimed at preventing disclosure of information and any repercussions resulting from loss of data confidentiality.

The study’s contribution lay in evaluating effectiveness of MMIs as a selection method for the medical program in the Brazilian socio-cultural context. Additionally, if personality profile differences among students with higher scores were found, the study results could help improve MMIs. The goal is not to favor individuals with specific personality profiles but to promote diversity by selecting students with well-developed socio-emotional skills.

## RESULTS

### Sample characteristics

The final sample comprised 225 candidates, with females accounting for 63%, and mean age of the candidates (aged between 18 and 34) being 21 years. Further, 47% of the sample had graduated 1 or 2 years earlier, while only 5.8% (n=13) had graduated high school the same year as when the evaluation was conducted ( Table 1 ).

**Table 1:** Baseline characteristics of the study participants

Total number of candidates	
Age (years)	
Mean (SD)	21 (3)
Range	18-34
Sex, n (%)	
Female	142 (63.1)
High school graduation, n (%)	
Year of survey	13 (5.8)
One year ago	61 (27.1)
Two years ago	45 (20.0)
Three years ago	37 (16.4)
Four years ago	27 (12.0)
Five or more years ago	42 (18.7)
Theoretical test (first phase)	
Mean (SD)	669 (98)
Range	91-759
MMI Scores (second phase)	
Mean (SD)	503 (119)
Range	55-786
Combined Scores	
Mean (SD)	601 (161)
Range	0-729
Admission process status, n (%)	
First call	63 (28.0)
Eliminated	10 (4.4)
Wait list	152 (67.6)

### Comparison of characteristics of candidates admitted and not admitted in the first call

The domains of neuroticism, extraversion, openness, and agreeableness showed no statistically significant differences between the groups, indicating that these variables did not influence final performance.

However, some differences were noted, especially among admitted candidates, in a few facets within these domains, such as Assertiveness (in Extraversion, p=0.083), and Ideas (in Openness, p=0.056), although they did not reach the stipulated 5% significance level.

**Figure 1:**
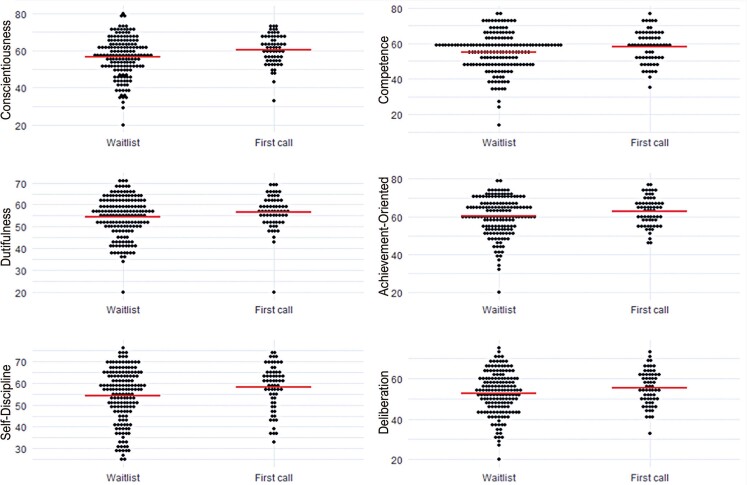
Dot plots of T-Scores between the Waitlisted and First Call Groups across different Conscientiousness facets. The red line represents the mean. The order facet was omitted due to non-significant differences between the groups

Conscientiousness was the only Big Five domain in which significant differences (p=0.004) were observed among candidates, with admitted candidates scoring higher. This effect was significant in four of its six facets: Competence (p=0.046), Dutifulness (p=0.043), Achievement-Oriented (p=0.050), and Self-Discipline (p=0.028), as can be seen in table 2 . However, despite the statistically significant differences found between the groups, the magnitude of these differences is small, as can be observed in figure 1 .

**Table 2:** Comparison of T-Scores for NEO PI R Conscientiousness Domain between First Call Admitted and Waitlisted Groups, Means and standard deviations (SD)

Facets	Total (n=225)	Waitlisted (n=162)	First call (n=63)	p value
Conscientiousness	57.7 (10.1)	57 (11)	60 (8)	0.004
Competence	55.9 (10.5)	55 (11)	58 (9)	0.046
Orderliness	55 (10.5)	54 (11)	57 (9)	0.163
Dutifulness	55 (8.8)	54 (9)	57 (8)	0.043
Achievement-Oriented	61 (9)	60 (10)	63 (8)	0.050
Self-discipline	55 (12)	54 (12)	58 (10)	0.028
Deliberation	53 (10)	53 (10)	55 (8)	0.080

### MMI results and Big Five correlations

A direct analysis of the MMI results did not reveal any statistically significant correlations, as shown in table 3 . Although the domain of Openness (r=0.156) and certain facets such as Anxiety (r=0.134) and Assertiveness (r=0.140) had significant p-values, correlations were weak.

**Table 3:** Correlation between MMI performance and NEO PI-R domain facets

	R	p value
Neuroticism	0.087	0.193
Anxiety	0.134	0.045
Anger	0.075	0.208
Depression	0.083	0.212
Embarrassment	0.063	0.350
Impulsiveness	-0.021	0.751
Vulnerability	0.111	0.097
Extraversion	0.071	0.288
Warmth	0.085	0.205
Gregariousness	0.002	0.975
Assertiveness	0.140	0.036
Activity	0.033	0.623
Excitement-seeking	-0.034	0.613
Positive Emotions	0.030	0.650
Openness	0.156	0.019
Fantasy	-0.018	0.792
Aesthetics	0.090	0.179
Feelings	0.051	0.443
Actions	0.101	0.131
Ideas	0.129	0.054
Values	0.112	0.094

## DISCUSSION

Critics have questioned the use of interviews in candidate selection processes citing a potential disruption of the fairness and homogeneity of the process. The risk of bias, for instance, prioritizing extroverts and underestimating introverts, could undermine the fairness of the process and lead to selecting candidates not based on the most desirable attributes. However, from the analysis conducted here, the MMI has proven to be an appropriate tool for selecting those best prepared for admission to medical school.

The comparison between candidates admitted in the first call and those not admitted consistently revealed the importance of the Conscientiousness domain and its facets as primary traits associated with higher performance in the final selection of candidates.

This result strengthens the hypothesis that effort, dedication, discipline, and organization are qualities crucial for success in an academically rigorous environment. This notion aligns with findings from recent literature, where high Conscientiousness translates into the ability to plan, execute, and maintain tasks, thereby ensuring student reliability. These characteristics are highly valued in intellectually demanding and professionally rigorous careers such as medicine.

Reviewing the body of research that objectively analyzes personality in the context of MMIs and university admissions, a previous study with 152 participants dismissed the notion of correlations between the Big Five traits and final outcomes, challenging the discriminatory effectiveness of interviews and tools like the NEO PI-R.^23^

However, another Australian study, with a more robust design including 868 subjects, confirmed the findings from this study in the Conscientiousness domain, as well as in Extraversion, justifying its plausibility as a complementary quality for positive professional interaction and medical practice, possibly because of its innate qualities of proactiveness, assertiveness, and adaptability in a naturally collaborative work environment.^22^

These findings reinforce the importance of considering personality aspects in the candidate selection process. A purely theoretical-content-based evaluation cannot lead to a truly comprehensive decision about an individual. This would overlook core aspects of their skills and functionality, which are crucial in determining their suitability for the institution’s model and values.

Therefore, a selection process would be far more successful if it could lead to identifying those who are more likely to possess greater resilience and show development when faced with the intrinsic challenges of the course. The result is a student who evolves and meets the institution’s expectations and needs, enriching it as a center for teaching and training.

The other domains of the Big Five do not have any influence on the outcome; this supports the view that MMIs can be a comprehensive and viable evaluation method. Neuroticism and Extraversion, which are conceptually slightly opposing domains, did not prove to be significant for differentiating students. The distribution across their spectrum did not show any statistical relevance, indicating that sensitivity to negative and positive emotions, respectively, may not necessarily be the hallmark of a good student. These are tangential, non-deterministic characteristics, each bringing its own dualities (good and bad aspects), thus worthy of equal regard during the selection process.

This impartiality toward any specific personality spectrum, while still aiming to form a mature and high-achieving student body, highlights the effectiveness of MMIs in preserving both individual and collective diversity.

Study limitations may have affected the analysis. Measurement bias could arise from the participants’ personalities, occurring systematically due to individuals’ propensity to respond to the questionnaire based on their personality type. Those in the higher percentiles of the Conscientiousness spectrum tend to be more diligent and committed, making them more likely to participate in the study. Conversely, extroverted individuals, who are more impulsive and carefree, or neurotic individuals, who are more anxious or worried, might be less likely to complete the personality assessment for various reasons.

Further, intentional manipulation of responses by candidates is another possibility, as the data were collected during the final stages of the selection process while they awaited their results. Although speculative, candidates may have minimized traits that might create a negative impression or exaggerated others to present themselves more favorably.

Furthermore, these findings have future applications that can be explored throughout the course. Understanding each student’s individuality allows personalized interventions to improve certain deficient characteristics.

Personality traits are not fixed and immutable; targeted efforts can improve them. For instance, exercises can be designed to (a) foster empathy in individuals scoring low on Agreeableness or (b) stimulate creativity and abstraction in those scoring low on Openness. Implementing strategies to develop specific skills that are not naturally strong in an individual enhances their functionality and optimizes their performance and well-being during medical training.

Stratification of students within a class also provides secondary benefits. A class structure that uses a collective and interactive learning model such as Team-Based Learning (TBL) relies on group separation as a central point of academic experiences. Defining groups with individual diversity ensures students are exposed to different reasoning models, problem-solving approaches, and socio-emotional interactions. This exposure to diverse perspectives fosters the exchange of new knowledge, broadens worldviews, and enhances general competencies and teamwork capabilities.

## CONCLUSION

The findings of this study underscore the value of using MMI as an effective selection tool for medical school admission. They indicate how MMI provides a balanced assessment that does not unduly favor or prove disadvantageous to candidates by disregarding irrelevant traits. The Conscientiousness domain significantly correlated with higher performance in MMIs, thus aligning with the hypothesis that its facets are central to achieving success in an academically rigorous and professionally demanding field such as medicine.

In other words, this study provides valuable insights into the effectiveness of MMIs in selecting candidates for medical school based on their socio-emotional competencies. By examining the relationship between personality traits and MMI performance, the study contributes to the refinement of selection methods, ensuring a diverse and competent student body that can enhance the quality of medical education and ensure the development of a resilient medical workforce.

In conclusion, this supports the ongoing integration of MMIs into admission processes while emphasizing the need for a comprehensive evaluation that includes both cognitive and non-cognitive attributes.
