# Supplementary material for: Effectiveness of Multiple Mini Interviews in medical school admissions: assessment using The Big Five Personality framework
Source: Einstein (Sao Paulo). 2025 Apr 25;23:eAO1352. doi: 10.31744/einstein_journal/2025AO1352 (PMC12094676; doi:10.31744/einstein_journal/2025AO1352)
Supplement: Supplementary file 1 [file 2317-6385-eins-23-eAO1352-suppl01.pdf]

Appendix 1. Socio-Demographic Questionnaire

1. What is your age as of December 31, 2020?
   (1) 17 years or younger
   (2) 18 years
   (3) 19 years
   (4) 20 years
   (5) 21 to 24 years
   (6) 25 years or older
2. What is your marital status?
   (1) Single
   (2) Married
   (3) Widowed
   (4) Separated
   (5) Divorced
   (6) Other
3. In which state does your family live? (Note: Do not indicate your temporary residence)
   (1) São Paulo
   (2) Minas Gerais
   (3) Paraná
   (4) Rio de Janeiro
   (5) Mato Grosso do Sul, Mato Grosso, Goiás, and Federal District
   (6) Other states or other countries (abroad)
4. Where does your family reside?
   (1) In the metropolitan region of São Paulo
   (2) In the countryside of São Paulo State
   (3) On the São Paulo State coast
   (4) In the capital of another state
   (5) In the countryside of another state
   (6) On the coast of another state
5. What type of high school course have you completed or will complete soon?
   (1) Regular
   (2) Technical
   (3) Adult education (Supletivo)
   (4) Other
6. What type of schooling did you undergo?
   (1) Entirely public school
   (2) Entirely private school
   (3) Mostly public school
   (4) Mostly private school
7. Have you ever attended or are currently attending any preparatory courses?
   (1) No
   (2) Yes, for less than one semester
   (3) Yes, for one semester
   (4) Yes, for one year
   (5) Yes, for more than a year
8. How many times have you taken the university entrance exam?
   (1) Never
   (2) Once
   (3) Twice
   (4) Thrice
   (5) Four or more times

9. What is your father's education level?
   (1) Illiterate
   (2) Incomplete elementary education
   (3) Completed elementary education
   (4) Completed high school
   (5) Incomplete higher education
   (6) Completed higher education
10. What is your mother's education level?
   (1) Illiterate
   (2) Incomplete elementary education
   (3) Completed elementary education
   (4) Completed high school
   (5) Incomplete higher education
   (6) Completed higher education
11. What is the total monthly income of your family? (Consider the total of the salaries of all family members)
   (1) Less than half of minimum wage (MW)
   (2) From half to 2x MW
   (3) From 2x MW to 3x MW
   (4) From 3x MW to 5x MW
   (5) From 5x MW to 10x MW
   (6) From 10x MW to 17x MW
   (7) Above 17x MW
12. How many people rely on the family income indicated in the previous question?
   (1) One
   (2) Two
   (3) Three
   (4) Four
   (5) Five
   (6) Six or more
13. How did you learn about the university entrance exam?
   (1) Family member/acquaintance
   (2) Your high school
   (3) While doing your preparatory course
   (4) Internet/social media
   (5) Other

Appendix 2. NEO PI-R Questionnaire

Graduate your answer with one of the following options: Strongly disagree, Disagree, Neutral, Agree, Strongly Agree.

I am a carefree person.

I genuinely like most people I meet.

I am a highly imaginative person.

I tend to distrust others' intentions.

I am known for my prudence and good judgment.

I am often annoyed by the way people treat me.

I dislike crowds and therefore avoid them.

I do not give much importance to matters concerning aesthetics and art.

I am not deceitful or cunning.

I prefer to keep my options open rather than plan everything in advance.

I rarely feel lonely or sad.

I am dominant, firm, and assertive.

I would not enjoy life if I did not have strong emotions or passions.

Available from: https://atosoficiais.com.br/lei/elaboracao-de-documentos-escritos-produzidos-pelo-psicologo-decorrentes-de-avaliacao-psicologica-cfp?origin=instituicao
